# Novel Reassortants of Oropouche Virus (OROV) Are Causing Maternal–Fetal Infection During Pregnancy, Stillbirth, Congenital Microcephaly and Malformation Syndromes

**DOI:** 10.3390/genes16010087

**Published:** 2025-01-15

**Authors:** David A. Schwartz

**Affiliations:** Perinatal Pathology Consulting, 490 Dogwood Valley Drive, Atlanta, GA 30342, USA; davidalanschwartz@gmail.com

**Keywords:** Oropouche virus, Oropouche fever, orthobunyavirus, vertical infection, reassortant, arbovirus, epidemic, fetal death, miscarriage, malformation syndrome, microcephaly, Brazil, congenital infection

## Abstract

Oropouche virus (OROV) is an orthobunyavirus endemic in the Brazilian Amazon that has caused numerous outbreaks of febrile disease since its discovery in 1955. During 2024, Oropouche fever spread from the endemic regions of Brazil into non-endemic areas and other Latin American and Caribbean countries, resulting in 13,014 confirmed infections. Similarly to other orthobunyaviruses, OROV can undergo genetic reassortment events with itself as well as other viruses. This occurred during this current outbreak, resulting in novel strains with increased pathogenicity and levels of transmission. For the first time, pregnant women with Oropouche fever have sustained poor perinatal outcomes, including miscarriage, fetal demise, stillbirths and malformation syndromes including microcephaly. In July 2024, PAHO issued an Epidemiological Alert warning of the association of OROV with vertical transmission. OROV has now been identified in the fetal blood, cerebrospinal fluid, placenta and umbilical cords, and fetal somatic organs including the liver, kidneys, brain, spleen, heart, and lungs using nucleic acid and antigen testing. Perinatal autopsy pathology has confirmed central nervous system infection from OROV in infants with congenital infection including microcephaly, ventriculomegaly, agenesis of corpus callosum, and neuronal necrosis. The latest data from Brazil show 3 confirmed cases of OROV vertical transmission; 2 cases of fetal death; 1 case of congenital malformation; and ongoing investigations into the role of OROV in 15 cases of fetal death, 3 cases of congenital malformations and 5 spontaneous miscarriages. This Commentary discusses the mechanisms and significance of development of novel reassortant strains of OROV during the current outbreak and their recent recognition as causing vertical infection and adverse perinatal outcomes among pregnant women with Oropouche fever.

## 1. Introduction

Oropouche fever is a neglected tropical disease caused by the arbovirus Oropouche virus (OROV) [1]. Initially discovered in Trinidad in 1955, the major arthropod vectors of Oropouche fever are biting midges (*Culicoides paraensis*), although mosquitos (*Culex quinquefasciatus*) can also transmit the virus [2,3]. OROV is endemic in South America and the Caribbean, where it circulates in both a sylvatic as well as urban cycle [4,5]. The region of highest endemicity for OROV infection is Brazil, where it has resulted in repeated outbreaks in jungle and forested regions such as Amazonas [6,7]. Oropouche fever typically results in a self-limited, mild disease, with headache, fever, chills, muscle and joint pain, nausea, vomiting, rash and photophobia, but in the minority of cases more severe symptoms can occur. These include central nervous system manifestations such as meningitis and encephalitis [6]. Since its initial discovery there have been over 30 outbreaks and greater than 500,000 cases of Oropouche fever in Latin America. It is likely that the true number of OROV infections is actually much higher as many cases remain undiagnosed, and others are misdiagnosed because of the similarity of Oropouche fever symptoms with viral infections such as dengue, West Nile, Zika, Mayaro, chikungunya, guama and yellow fever [6,7,8]. Molecular virology data indicate that beginning in late 2022 or 2023 in Amazonas in Northern Brazil, OROV rapidly increased in prevalence in areas of the country with known endemicity, subsequently spreading throughout Brazil into regions that had not been endemic including Mato Grosso, Minas Gerais, Bahia, Mato Grosso do Sul, Santa Catarina, Ceará, Piauí, Pernambuco, Espírito Santo, and Rio de Janeiro [7,9]. OROV continued to increase in prevalence in areas with known endemicity in Brazil in late 2023 and subsequently spread further throughout Brazil and Latin American countries where the infection had not been previously detected [10,11]. These new areas of infection not only included rural regions but also urban centers in Peru, Colombia, Cuba and Bolivia, where sustained transmission developed. Between 1 January and 13 December 2024, there were 13,014 confirmed cases of OROV infection identified, most from Brazil (10,940, with two deaths), and the remainder from Colombia (*n* = 74), Bolivia (*n* = 356), Cuba (*n* = 603), Ecuador (*n* = 3), Guyana (*n* = 2), Peru (936), Canada (*n* = 2 imported) Cayman Islands (*n* = 1 imported) and the Dominican Republic [10,11]. Imported cases from infected travelers have occurred in the United States (94 imported cases) and Europe (30 cases) [11].

The current Oropouche fever outbreak has resulted in new features of morbidity and mortality that are associated with the spread of novel reassortment variants of OROV. For the first time, Oropouche fever has been associated with adult fatalities, causing the death of two non-pregnant women who had no co-morbidities [12]. Significantly, during this recent outbreak there have been multiple reports of maternal–fetal OROV infection associated with poor obstetrical outcomes including miscarriage, stillbirth, and congenital fetal malformations including microcephaly [12,13,14,15,16]. The occurrence of these adverse pregnancy outcomes is a worrisome phenomenon that is currently undergoing intense investigation. This Commentary discusses these obstetrical and fetal events in view of the virology of OROV and the novel genetic strains that are causing the current outbreak.

## 2. Virology of OROV

OROV is a bunyavirus belonging to the Simbu serogroup of the genus *Orthobunyavirus*, order *Bunyavirales*, family *Peribunyaviridae* [17,18]. Bunyaviruses are characterized by having a negative-sense, single-stranded RNA structure and lipid envelopes within a spherical virion, and a viral surface possessing a continuous layer of glycoproteins that are believed to have a function in creating new virions by budding from the host cell membrane. The envelope of OROV originates from the host cell membrane and is covered by spikes formed by two type 1 transmembrane glycoproteins, Gn and Gc [19]. Unlike many other arboviruses, the genome of OROV is tripartite, consisting of three single-stranded negative sense helical segments of genomic RNA that are termed small (S), medium (M), and large (L) based upon their molecular sizes [18,19,20]. The L segment contains the genetic information-encoding RNA-dependent RNA polymerase (RDRP), an enzyme which is necessary for viral replication, synthesizing both sense and antisense RNA within the cytoplasm of infected cells. The M segment encodes a precursor polyprotein which produces OROV surface glycoproteins Gn and Gc, as well as a nonstructural protein, NSm. The precise function of the NSm protein is not completely understood but is thought to be involved in viral assembly and budding processes. The segmentation of the OROV genome permits genetic reassortment events to occur [2,4,5,21,22,23]. The S segment encodes for production of the viral nucleocapsid protein (N) and another non-structural protein, NSs, from overlapping open reading frames [21,22]. The N protein has an important function in both viral replication and transcription through maintaining the integrity of the viral genome [19]. It does this by forming the nucleocapsid through its interaction with viral RNA and enveloping and protecting the viral genomic segments. The NSs protein plays an important role in the avoidance of the host antiviral response. Although it does not contribute to replication or the viral protein coating, the NSs protein acts as a virulence factor by inhibiting interferon response from the host, thus increasing viral replication in infected cells [19,21,23]. Located adjacent to the coding regions on each segment are untranslated regions that serve a role in viral packaging, replication and transcription [21,24].

Four OROV genotypes have traditionally been designated using phylogenetic analysis of the N gene, with an average nucleotide difference among the four genotypes measuring five percent [24,25,26]. The geographic distribution of these genotypes includes Genotype I reported from Maranhão, Acre, Pará and Amazonas, as well as French Guiana. Genotype II is found in Amapá, Brazil. Genotype III occurs in Pará, Brazil, and Panama, and Genotype IV has been reported in Rondônia, Brazil [27]. There are more recent classification models that are based on the genetic sequences of all three RNA segments that propose only two clades or lineages within the M or L segment phylogenies [24].

## 3. Genetics of OROV

An important genetic feature of orthobunyaviruses is their segmented genomes, enabling them to undergo rapid reassortment when two genetically related viruses infect the same cell [21,28]. This capability for reassortment is a powerful mechanism in the evolution of bunyaviruses, in which new viral strains and species with novel properties can be naturally created [22,28]. These new properties can include increased virulence and other pathogenic features. This is especially true among the Simbu serogroup of bunyaviruses, to which OROV belongs. The Simbu serogroup contains 33 named viruses grouped into 19 virus species [29]. Genetic reassortment has been identified in several orthobunyaviruses including Potosi virus (POTV), Iquitos virus (IQT), Ngari virus (NRIV), Aino virus (AINOV), Perdoes virus (PEDV), Madre de Dios virus (MDDV), Apeu virus (APEUV) and Fort Sherman virus (FSV); it has also been produced experimentally between OROV and orthobunyaviruses in the Simbu subgroup [21,30]. The newly created reassorted viruses typically contain the S and L segments from the parental strain, and in most cases the donor of the M segment is not known [21]. Examples of naturally occurring OROV reassortment events that create new bunyaviruses include Iquitos virus. IQT was first isolated in the Peruvian Amazon region from a febrile patient and resembles OROV in the S and L segments but possesses a novel Simbu serogroup M segment, and is serologically distinct from OROV [24,31]. PDEV and MDDV share L and S segments with OROV, but their M segments are derived from as of yet unidentified orthobunyaviruses [21].

## 4. Novel Reassortant Strains Spreading in the 2023–2024 OROV Outbreak

Following the original description of OROV from a febrile forest worker in 1955 in Trinidad and Tobago, the virus has been responsible for multiple local outbreaks that have infected many hundreds of thousands of persons throughout Latin America, with most occurring in the Amazonian states of Brazil [32]. The first recognized OROV outbreak occurred in Belém, Brazil in 1961 and affected approximately 11,000 persons, with seven additional outbreaks of OROV also occurring in Pará State during the same time period. In 1980 and 1981 the two largest outbreaks of Oropouche fever, during which greater than 100,000 people were infected, occurred in Belém, Pará State, and in Manaus, Amazonas State [33]. After those outbreaks and extending to 2005 there were only sporadic cases or self-limited outbreaks reported in the Brazilian Amazon regions, mainly in small villages, which suggested there was ongoing silent circulation of the virus [18,19,33,34]. Over 30 outbreaks of Oropouche fever had been recorded in Brazil and other Latin American countries prior to the 2023–2024 outbreak, making it the second most common arboviral infection to occur in Brazil.

Between November 2023 and August 2024 there was a rapid and significant increase in Oropouche fever cases in Brazil as well as in Bolivia, Colombia, and Peru. The number of persons developing OROV fever became so great during this outbreak that there was a 58.8-fold higher incidence of infection than the annual median between 2015 and 2023 [35]. Understanding the reasons behind the rapid spread of Oropouche fever throughout Latin America and the Caribbean since late 2023 has remained problematic, but it was likely a result of a combination of different events and circumstances. Factors that have been implicated as partially accounting for this explosive spread of infection include climate change with an increase beyond normal rainfall during the 2023–2024 El Niño event, manmade modifications of the habitat including increased agricultural activity, cattle ranching, and widespread deforestation, and human and animal mobility factors [36]. Because Oropouche fever is an arbovirus spread to humans via blood meals from arthropods including the biting midge (*Culicoides paraensis*) and mosquitos (*Culex quinquefasciatus*, *Aedes aegypti*, *Ochlerotatus serratus*), deforestation may be an important contributing factor in the spread of infection, due to the altering of the natural habitat of OROV and its vectors. A greater prevalence of OROV-vector–human interactions has resulted from higher human population density due to progressive urbanization [37].

However, the most significant factor for the explosive expansion of the outbreak appears to be genetic reassortment producing new strains of OROV (Table 1) [2,7,35]. These events appear to have initially arisen in the Amazon basin region of Brazil. Based upon molecular virology data, Naveca et al. [38] have proposed that the initial outbreak probably began in late 2022 or early 2023. This wave was followed in October 2023 by a more significant wave of infections that eventually reached its zenith during the Amazon rainy season, possibly as a result of increased insect vector density [38]. Analysis of the genomes of OROV isolates during the past several years has shown a rapid north-to-south movement from the original location of the virus in the Amazon Basin region to areas that had not been endemic for the infection [39].

The ongoing Oropouche fever outbreak is probably due to emergence of novel OROV strains that had their origins following complex reassortment events involving diverse S, M and L segments (Table 1) [7,35,40]. The complexity of OROV recombinant events associated with this outbreak was examined by Naveca et al., who sequenced 382 clinical OROV isolates from three Brazilian states between August 2022 and February 2024 and compared them with 72 OROV sequences from the period 1955 to 2021 saved in GenBank (NCBI) [38]. Their results indicated that the OROV isolates currently in circulation possess an M segment that is closely related to the most prevalent lineage in Brazil, and this was termed lineage 1. In contrast, they found that the L and S segments branch more closely with the 2008 and 2021 sequences isolated from Peru, Columbia and Ecuador, and termed this lineage 2 [38]. De Melo Iani et al. identified 21 reassortment events occurring among 133 whole genome sequences, including analysis of the L, M and S segments, and believed that this was indicative of possible viral adaptation to new environments [39]. Full-length genomic analyses of many hundreds of OROV isolates from the current outbreak coincide with the development of a new genetic reassortments [39]. One new lineage, termed BR-2015–2024, was first identified in Colombia containing the M segment of viruses detected in the eastern Amazon region (2009–2018) and the L and S segments of viruses detected in Peru, Colombia and Ecuador (2008–2021) [32]. Examining two genomic sequences from 2023 to 2024 epidemic isolates—designated AM0059 and AM0088—obtained from patients with Oropouche fever in Amazonas State, Scachetti et al. [35] lead to the identification of a novel reassortment involving an M segment from a previously circulating OROV strain in northern Brazil together with L and S segments from a prior reassortment that was derived from the Iquitos virus. Their analysis also demonstrated a median amino acid similarity greater than 99.9965% between OROV strains AM0059 and AM0088 and all three genomic segments of 390 reassortant OROV strains that had been isolated from Brazil, Peru, and Italy during 2023 and 2024 [35]. Significantly, these OROV reassortment strains demonstrated much higher replicative competence in cultured mammalian cells in comparison with the ancient OROV strain BeAn19991m and also produced a greater number and size of necrotic plaques following inoculation onto a cell monolayer [35], indicating increased virulence. In addition to these viral strains from infected patients in South America, the whole genome of an OROV strain imported into Europe was recently analyzed [39]. This strain, which was isolated from a patient with Oropouche fever acquired in Cuba, was most closely related to an OROV sequence from the 2020 French Guiana outbreak, possessed a reassortment involving the S and L segments that had great similarity with sequences belonging to a new cluster (OROV_SCDC_2024), with the M segment having marked similarity with older sequences [39].

These studies all indicate that the current OROV outbreak in Latin America and the surrounding regions is the result of novel recombinant viral strains having distinct genetic features (Table 1) [19,35,38,39,40,41,42,43].

**Table 1:** Reassortant strains of Oropouche virus (OROV) circulating during the 2023–2024 Oropouche fever outbreak.

OROV Reassortant Id	Location of Isolate	Isolation Date	Host	References
AM0059	Manaus, Brazil	2024	Human	[35]
AM0088	Brazil	2024	Human	[35]
BR-2015–2024	Amazonas, Brazil	2010–2014	Human	[38,42]
PE/CO/EC-2008–2021	Leticia, Colombia	2008–2021	Human	[32]
OROV_SCDC_2024	Cuba (from traveler to Italy)	2024	Human	[41]

## 5. OROV Infection in Pregnancy

The initial report of adverse pregnancy outcomes in women having Oropouche fever occurred during the first outbreaks of OROV infection from Amazonas State in 1980. Among a total of nine infected pregnant women, two had miscarriages during the second month of pregnancy [44]. There was no confirmatory evidence or follow-up investigation of a causal role for the virus in these events, and scant attention was paid to these data from the medical and public health communities. During the 2024 OROV outbreak, however, it became evident that OROV is the newest emerging virus to cause adverse pregnancy outcomes. Between January and July 2024 there had already been 7700 confirmed cases of Oropouche fever in Brazil and four other Latin American countries when the first suspected cases of mother-to-fetus viral transmission were reported among pregnant women with OROV infection [45]. On 12 July 2024, the Brazilian authorities reported to the Pan American Health Organization/World Health Organization (PAHO/WHO) the occurrence of a presumptive case of maternofetal (vertical) transmission of OROV [46] in Pernambuco State. The pregnant mother resided in Rio Formoso Municipality, an area of Northeastern Brazil where there had been OROV transmission occurring since May 2024. She developed Oropouche fever symptoms including headache, epigastric pain and fever on 24 May 2024 at 30 weeks gestation, and reported being in close contact with a person having OROV infection. Samples collected from the woman on 3 June had a reactive response for dengue and chikungunya viruses by IgM ELISA testing, and both maternal serum and the placenta tested positive for OROV using RT-PCR. The mother presented for medical attention on 6 June after noticing lack of fetal movement, and an intrauterine fetal demise was confirmed. Following delivery and performance of a fetal autopsy, molecular analysis performed at the Evandro Chagas Institute confirmed infection with OROV based upon positive RT-PCR testing of the umbilical cord blood and placenta, as well as multiple fetal somatic organs including the liver, kidneys, brain, spleen, lungs, and heart. Fetal tissue specimens were negative for other arboviruses including Zika, dengue, Mayaro, and chikungunya. The autopsy pathology and molecular virology findings from this case were characteristic of an intrauterine vertical infection, similar to the patterns of intrauterine transplacental transmission of recent emerging viruses that cause stillbirth such as SARS-CoV-2 [47,48,49], Zika virus [50] and mpox virus [51,52,53].

A second fetal demise suspected to be due to Oropouche fever occurred in a pregnant woman from Jaqueira, Pernambuco state [45,54]. She developed clinical features of OROV infection during the first trimester, developed uterine hemorrhage, and had a miscarriage on 27 June 2024, at 8 weeks gestation. Maternal serum collected on 12 June tested positive for OROV by PCR and negative for other arboviral pathogens, and was reactive for dengue virus using IgM ELISA.

On 17 July, PAHO issued an Epidemiological Alert regarding the association of OROV with vertical transmission, miscarriage, fetal deaths and congenital fetal anomalies, asking that Member States increase surveillance for development of maternal–fetal transmission in their territories and to report these events [45]. As part of this announcement, it was stated that the Brazil IHR National Focal Point reported that in June 2024 the Brazilian IEC had investigated samples of cerebrospinal fluid and serum collected during a previous arbovirus study that had tested negative for dengue, chikungunya, Zika, and West Nile virus. This further analysis revealed there were four newborns having microcephaly (three newborns at 1 day of life and one at 27 days of life) that had IgM antibodies against OROV in serum samples (one newborn at 1 day of life and another at 27 days of life) and cerebrospinal fluid (two newborns at 1 day of life and a newborn at 27 days of life, in which IgM was also detected in cerebrospinal fluid) [46,54].

Following this announcement, on 24 July 2024, a 40-year-old pregnant gravid 3 para 1 woman from Ceará, Brazil developed chills, fever, severe headache and generalized myalgia at 30 weeks and 3 days gestation [55]. She had gestational diabetes treated with Metformin and a past medical history of a first-trimester pregnancy loss. Obstetrical ultrasounds performed on four occasions had been normal. She sought medical attention on 27 July for a dark vaginal discharge accompanied by light vaginal bleeding. An ultrasound revealed a living fetus with macrosomia (EFW > 97.5 percentile), a normal heart rate, normal amniotic fluid volume, and a posterior placenta having a heterogeneous Grade I echotexture without signs of pathological alterations. On 5 August she presented with a complaint of decreased fetal movement since 31 July and continued fever and light vaginal bleeding. Obstetrical ultrasound confirmed intrauterine fetal demise. Maternal blood that was obtained at the initial evaluation demonstrated acute OROV infection by molecular testing, and which was negative for dengue, Zika, chikungunya, and Mayaro viruses. The patient stayed in the Maternity Ward for 48 h following the diagnosis of fetal demise in an attempt to induce labor, but eventually a cesarean section had to be formed. Because the family refused a full autopsy, a minimally invasive autopsy was performed. The stillborn 2190 g macerated male fetus showed no malformations. OROV RNA was detected in multiple fetal visceral organs including the liver and lungs. The placenta weighed 354 g (10th percentile) and grossly showed areas of infarction affecting approximately 30 percent of the maternal surface [56]. Both the placenta and umbilical cord tested positive for OROV, supporting intrauterine transplacental OROV transmission. The fetal brain and cerebrospinal fluid were also positive for OROV, indicating central nervous system infection as is seen in other bunyaviruses [57]. RT-PCR testing for other arboviruses including Mayaro, dengue, chikungunya, and Zika viruses were negative in all specimens. Unfortunately, pathological interpretation of the fetal autopsy tissues was limited by autolysis, but in the placenta there were abnormal microscopic findings of increased syncytial knots, reduced intervillous space and frequent areas of fibrin deposition indicating malperfusion. In order to examine the relationship between the OROV viral strain infecting this fetus and the new reassortment variants of OROV that had emerged in Brazil, the authors employed an amplicon-based whole-genome nucleotide sequencing protocol using liver, umbilical cord and brain tissues from the stillborn infant. The resulting combined genomes clustered in a highly supported monophyletic subclade within the major clade originating from Amazonas state, confirming that the OROV strains were linked [42].

In October 2024, a group of Brazilian researchers published regarding a series of newborns having congenital microcephaly and other malformations which were all associated with pregnant mothers having OROV infection [15,16,57]. The study groups consisted of a cohort of 65 historical cases of microcephaly, arthrogryposis, and other congenital malformations lacking a known cause dating to between 2015 and 2021, and a group of 3 cases of individuals born in 2024, in ten states in Brazil. Three historical cases involving congenital anomalies included positive tests for OROV, and the three cases of individuals born in 2024 all involved positive tests for OROV as well. One of the newborns died at Day of Life 47 and underwent an autopsy with tissues tested by histopathology, immunohistochemistry, and real-time RT-PCR. The autopsy revealed significant central nervous system pathology both macroscopically and microscopically, including microcephaly, neuronal necrosis, apoptotic changes of astrocytes, microglia and neurons, vacuolization, and tissue atrophy. OROV RNA was identified by molecular pathology in the lungs, kidney, brain, and pleural and central nervous system fluids. Antigens of OROV were found in the liver, kidney, heart, and lungs, as well as in the central nervous system in neurons, microglia and endothelial cells, suggesting vasculitis [15].

The most recent WHO epidemiological report of adverse perinatal outcomes associated with Oropouche fever in Brazil from 5 December 2024 includes three confirmed cases of OROV vertical transmission; two cases of fetal death, one in Pernambuco and one in Ceará; and one case of congenital malformation in Acre. Investigation is continuing into 15 cases of fetal death in Pernambuco, 3 cases of congenital malformations in Acre (2 cases) and Bahia (1 case), and 5 spontaneous miscarriages in Pernambuco [58].

In Cuba, 7 pregnant women having Oropouche fever have been identified, among whom 2 delivered liveborn infants with no congenital abnormalities [11]. Three newborns with central nervous system congenital abnormalities have been identified which are suspected to have an infectious etiology. One of these 3 infants was diagnosed with OROV in fetal cardiac blood, and the other 2 are under investigation [58].

It remains uncertain if Oropouche fever is more severe when occurring during pregnancy [59,60,61]. The clinical symptoms of OROV infection in pregnant women appear to be the same for both pregnant and non-pregnant women. Information is currently unavailable for the recommendation of optimal timing for initial fetal ultrasonography in pregnant women with Oropouche fever. The role of amniocentesis in the diagnosis of intrauterine fetal infection with OROV is unknown. Oropouche fever during pregnancy is diagnosed using the same methods as for non-pregnant individuals. These tests include the plaque reduction neutralization test (PRNT); if a pregnant woman is positive by PRNT, collection of acute and convalescent serum samples is indicated, which should be tested for a four-fold increase in neutralizing antibodies. Real-time reverse transcription–polymerase chain reaction (RT-PCR) can detect OROV RNA in serum, cerebrospinal fluid (CSF) or solid tissues such as the placenta or fetal organs [59,60,61]. No specific antiviral medication or vaccine is available for prevention or treatment of Oropouche virus disease, or for preventing vertical transmission and congenital fetal abnormalities.

## 6. Potential Pathogenesis of Vertical Infection from OROV Reassortant Strains

**Figure 1:**
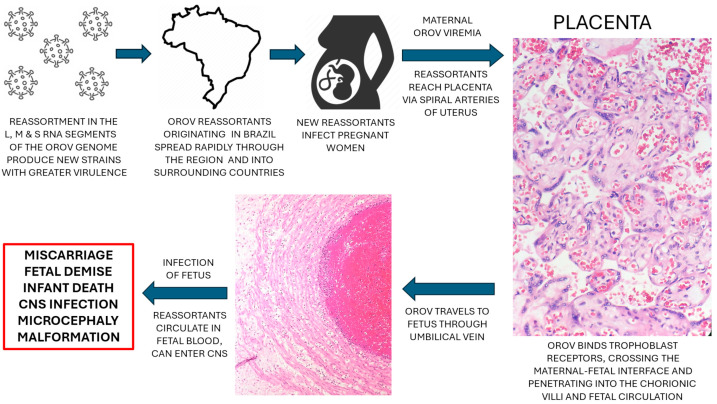
Proposed pathogenesis of vertical infection from new Oropouche virus (OROV) reassortant strains. OROV reassortants have greater virulence than previous strains, produce viremia in infected pregnant women and can cross the maternal–fetal interface to infect the placenta and fetus.

Transplacental transmission of OROV is most likely dependent on an episode of maternal viremia, which occurs in many viruses which cause maternal–fetal transmission, including other bunyaviruses, SARS-CoV-2 and Zika virus [12,57]. A potential mechanism involves the entry of OROV into the placenta through the uterine spiral arteries, where it circulates in the maternal intervillous space and is exposed to the syncytiotrophoblast, the major protective cell layer at the maternal–fetal interface (Figure 1). Following binding to a yet unidentified surface receptor on the syncytiotrophoblast, OROV may be internalized into the trophoblast, enter the villous stroma and potentially Hofbauer cells (villous stromal macrophages), and eventually infect and/or penetrate villous capillary endothelial cells, thereby reaching the villous circulation of the fetus. OROV can then travel through the chorionic vessels to the umbilical vein, eventually reaching the fetal somatic circulation. Upon entering the fetal brain, OROV can infect neurons and glial cells and produce the central nervous system abnormalities already described in some congenitally infected fetuses. Bunyaviruses of the Simbu serogroup are known to have a predilection for causing central nervous system infections in human and animals, including Akbane virus (AKAV/AV), Schmallenberg virus (SMV), Aino virus (AINOV), and Peaton virus (PEAV) [57]. Future analysis of infected placentas using immunohistochemistry and nucleic acid hybridization will undoubtedly reveal additional information on the placental cell types permissive to infection, such as syncytiotrophoblast, villous stromal and endothelial cells, Hofbauer cells, extravillous trophoblast and decidua that have been identified in other congenital viral infections.

## 7. Discussion

Oropouche fever has historically been medically significant as a neglected benign tropical disease that mimicked other arbovirus infections with which it shared geographic overlap including Zika, dengue, Mayaro, West Nile and chikungunya viruses. During the 2023–2024 surge of OROV infections that resulted from spread of novel reassortant strains that originated in Brazil, this concept of Oropouche fever as a mild, self-limited disease has changed. Two young women developed Oropouche and died, the first fatalities attributable to this arbovirus. Both women, aged 21 and 24 years, developed headache, fever, nausea, vomiting, myalgia, abdominal pain, diarrhea and retroorbital pain. The younger woman also developed arthralgia, red-colored rash, purple spots and spontaneous bleeding from the gums, nose, and vagina. These women had rapid disease progression with high viral loads (RT-qPCR assay cycle threshold values of 8 and 16). No neurological symptoms were observed in either case. Clinical reports note that both patients died 4 days after symptom onset from severe coagulopathy and liver impairment. Neither woman had any co-morbidities [7,45].

Oropouche fever has been estimated to be an infectious risk factor for as many as five million people throughout the Americas, making it one of the most important emerging viral diseases in Latin America [62]. Despite the occurrence of hundreds of thousands of cases of Oropouche fever in Latin America and the Caribbean since its initial recognition in 1955, the 2023–2024 outbreak from genetically reassorted viral strains has shown the first epidemiologically validated associations of OROV maternal infection with adverse perinatal events. There have been clinically, microbiologically and pathologically confirmed cases of intrauterine transplacental (vertical) infection occurring between mothers having Oropouche fever and the fetus [12]. Following the PAHO’s issuance of an Epidemiological Alert warning regarding the association of OROV with vertical transmission in July 2024 it has been confirmed that OROV can infect the placenta and fetus, causing congenital infection and adverse perinatal outcomes including spontaneous miscarriage, stillbirth, and congenital anomalies including nervous system involvement with microcephaly. As of 5 December 2024, there have been multiple reports of miscarriages, fetal deaths, and congenital anomalies associated with vertical OROV infection in Brazil [58]. In Cuba, three cases of congenital central nervous system anomalies associated with maternal OROV infection are being investigated, one of which had OROV in fetal heart blood [58]. Stillborn fetuses from mothers with Oropouche fever have demonstrated OROV nucleic acid by RT-PCR in the placenta, umbilical cord, spleen, liver, brain, kidneys, lungs, and heart. Autopsy of an infant who died at 47 days of life with congenital OROV infection had microcephaly, ventriculomegaly, agenesis of corpus callosum, and joint malformations, with pathology evidence necrosis of neurons, microglia and astrocytes and viral antigens in multiple organs. Placental pathology analysis has revealed multiple abnormalities in a fetus having congenital infection including findings of infarction and malperfusion [15]. Thus, OROV joins the list of TORCH (Toxoplasmosis, Other, Rubella, Cytomegalovirus, Herpes) infections, including such recent additions as Zika virus, Ebola virus, SARS-CoV-2 virus, and mpox virus, that can be transmitted vertically through the placenta, causing congenital infections, fetal morbidity and mortality.

The current outbreak of Oropouche fever reaffirms the importance of phylogenetic monitoring of the emergence of novel reassortant strains of orthobunyaviruses which may have enhanced transmission dynamics and pathogenicity. OROV has circulated in endemic areas for many decades, producing many hundreds of thousands of human infections without causing known loss of life or obstetrical complications, until the advent of new reassortant strains in Brazil. The sudden recognition of obstetrical complications associated with emergent reassortant strains of OROV, including numbers of reports of pregnancy losses, congenital vertical transmission, infection of the fetal central nervous system and malformation syndromes, highlights the importance of conducting intensive studies regarding the not only the potential mechanism of maternal–fetal transmission, but also its impact on obstetrical outcomes and fetal health [63].

## References

[1] Sakkas H., Bozidis P., Franks A., Papadopoulou C. (2018). Oropouche Fever: A Review. Viruses.

[2] Wesselmann K.M., Postigo-Hidalgo I., Pezzi L., De Oliveira-Filho E.F., Fischer C., De Lamballerie X., Drexler J.F. (2024). Emergence of Oropouche Fever in Latin America: A Narrative Review. Lancet Infect. Dis..

[3] Moreira F.R.R., Dutra J.V.R., de Carvalho A.H.B., Reis C.R., Rios J.S.H., Ribeiro M.O., Arruda M.B., Alvarez P., Souza R.P., Voloch C. (2024). Oropouche Virus Genomic Surveillance in Brazil. Lancet Infect. Dis..

[4] Centers for Disease Control and Prevention Clinical Overview of Oropouche Virus Disease. 25 October 2024. https://www.cdc.gov/oropouche/hcp/clinical-overview/index.html.

[5] Pinheiro F.P., Travassos da Rosa A.P., Travassos da Rosa J.F., Ishak R., Freitas R.B., Gomes M.L., LeDuc J.W., Oliva O.F. (1981). Oropouche virus. I. A Review of Clinical, Epidemiological, and Ecological Findings. Am. J. Trop. Med. Hyg..

[6] The Lancet Infectious Diseases (2024). Oropouche Fever, The Mysterious Threat. Lancet Infect. Dis..

[7] Tilston-Lunel N.L. (2024). Oropouche Virus: An Emerging Orthobunyavirus. J. Gen. Virol..

[8] Lorenz C., Azevedo T.S., Virginio F., Aguiar B.S., Chiaravalloti-Neto F., Suesdek L. (2017). Impact of Environmental Factors on Neglected Emerging Arboviral Diseases. PLoS Negl. Trop. Dis..

[9] Martins-Filho P.R., Carvalho T.A., Dos Santos C.A. (2024). Spatiotemporal Epidemiology of Oropouche Fever, Brazil, 2015–2024. Emerg. Infect. Dis..

[10] Anderer S. (2024). Oropouche Virus Spreads to New Regions in Latin America. JAMA.

[11] Pan American Health Organization Epidemiological Update Oropouche in the Americas Region—13 December 2024. https://www.paho.org/sites/default/files/2024-12/2024-dic-13-epialert-oropouche-eng-final.pdf.

[12] Schwartz D.A., Dashraath P., Baud D. (2024). Oropouche Virus (OROV) in Pregnancy: An Emerging Cause of Placental and Fetal Infection Associated with Stillbirth and Microcephaly following Vertical Transmission. Viruses.

[13] Martins-Filho P.R., Carvalho T.A., Dos Santos C.A. (2024). Oropouche Fever: Reports of Vertical Transmission and Deaths in Brazil. Lancet Infect. Dis..

[14] Garima S.K., Priyanka S.K.K., Srikanth P.T., Jitendra S.B. (2024). Silent Risk: The Vertical Transmission of Oropouche Virus. Eur. J. Clin. Microbiol. Infect. Dis..

[15] das Neves Martins F.E., Chiang J.O., Nunes B.T.D., Ribeiro B.F.R., Martins L.C., Casseb L.M.N., Henriques D.F., de Oliveira C.S., Maciel E.L.N., Azevedo R.D.S. (2024). Newborns with Microcephaly in Brazil and Potential Vertical Transmission of Oropouche Virus: A Case Series. Lancet Infect. Dis..

[16] Brazil Ministry of Health Ministério da Saúde Informa caso de Anomalia Congênita Associada à Oropouche. 8 August 2024. https://www.gov.br/saude/pt-br/canais-de-atendimento/sala-de-imprensa/notas-a-imprensa/2024/ministerio-da-saude-informa-caso-de-anomalia-congenita-associada-a-oropouche.

[17] Azevedo R.S., Nunes M.R., Chiang J.O., Bensabath G., Vasconcelos H.B., Pinto A.Y., Martins L.C., Monteiro H.A., Rodrigues S.G., Vasconcelos P.F. (2007). Reemergence of Oropouche Fever, Northern Brazil. Emerg. Infect. Dis..

[18] Travassos da Rosa J.F., de Souza W.M., Pinheiro F.P., Figueiredo M.L., Cardoso J.F., Acrani G.O., Nunes M.R.T. (2017). Oropouche Virus: Clinical, Epidemiological, and Molecular Aspects of a Neglected Orthobunyavirus. Am. J. Trop. Med. Hyg..

[19] Zhang Y., Liu X., Wu Z., Feng S., Lu K., Zhu W., Sun H., Niu G. (2024). Oropouche Virus: A Neglected Global Arboviral Threat. Virus Res..

[20] Riccò M., Corrado S., Bottazzoli M., Marchesi F., Gili R., Bianchi F.P., Frisicale E.M., Guicciardi S., Fiacchini D., Tafuri S. (2024). (Re-)Emergence of Oropouche Virus (OROV) Infections: Systematic Review and Meta-Analysis of Observational Studies. Viruses.

[21] Tilston-Lunel N.L., Hughes J., Acrani G.O., da Silva D.E., Azevedo R.S., Rodrigues S.G., Vasconcelos P.F., Nunes M.R., Elliott R.M. (2015). Genetic Analysis of Members of the Species Oropouche Virus and Identification of a Novel M Segment Sequence. J. Gen. Virol..

[22] Elliott R.M. (2014). Orthobunyaviruses: Recent Genetic and Structural Insights. Nat. Rev. Microbiol..

[23] Bridgen A., Weber F., Fazakerley J.K., Elliott R.M. (2001). Bunyamwera Bunyavirus Nonstructural Protein NSs is a Nonessential Gene Product That Contributes to Viral Pathogenesis. Proc. Natl. Acad. Sci. USA.

[24] Files M.A., Hansen C.A., Herrera V.C., Schindewolf C., Barrett A.D.T., Beasley D.W.C., Bourne N., Milligan G.N. (2022). Baseline mapping of Oropouche Virology, Epidemiology, Therapeutics, and Vaccine Research and Development. NPJ Vaccines.

[25] Vasconcelos H.B., Nunes M.R., Casseb L.M., Carvalho V.L., Pinto da Silva E.V., Silva M., Casseb S.M., Vasconcelos P.F. (2011). Molecular Epidemiology of Oropouche Virus, Brazil. Emerg. Infect. Dis..

[26] Dong J., Li Z., Gao S., Zhang L. (2024). A Bibliometric Analysis of Oropouche Virus. Front. Microbiol..

[27] Sah R., Srivastava S., Mehta R., Khan S.R., Kumar S., Satpathy P., Mohanty A., Ferraz C., Feehan J., Apostolopoulos V. (2024). Oropouche Fever Fatalities and Vertical Transmission in South America: Implications of a Potential New Mode of Transmission. Lancet Reg. Health Am..

[28] Tilston-Lunel N.L., Shi X., Elliott R.M., Acrani G.O. (2017). The Potential for Reassortment between Oropouche and Schmallenberg Orthobunyaviruses. Viruses.

[29] O’Connor T.W., Hick P.M., Finlaison D.S., Kirkland P.D., Toribio J.-A.L.M.L. (2024). Revisiting the Importance of Orthobunyaviruses for Animal Health: A Scoping Review of Livestock Disease, Diagnostic Tests, and Surveillance Strategies for the Simbu Serogroup. Viruses.

[30] Guagliardo S.A.J., Connelly C.R., Lyons S., Martin S.W., Sutter R., Hughes H.R., Brault A.C., Lambert A.J., Gould C.V., Staples J.E. (2024). Reemergence of Oropouche Virus in the Americas and Risk for Spread in the United States and Its Territories, 2024. Emerg. Infect. Dis..

[31] Aguilar P.V., Barrett A.D., Saeed M.F., Watts D.M., Russell K., Guevara C., Ampuero J.S., Suarez L., Cespedes M., Montgomery J.M. (2011). Iquitos Virus: A Novel Reassortant Orthobunyavirus Associated with Human Illness in Peru. PLoS Negl. Trop. Dis..

[32] Usuga J., Limonta D., Perez-Restrepo L.S., Ciuoderis K.A., Moreno I., Arevalo A., Vargas V., Berg M.G., Cloherty G.A., Hernandez-Ortiz J.P. (2024). Co-Circulation of 2 Oropouche Virus Lineages, Amazon Basin, Colombia, 2024. Emerg. Infect. Dis..

[33] Baisley K.J., Watts D.M., Munstermann L.E., Wilson M.L. (1998). Epidemiology of Endemic Oropouche Virus Transmission in Upper Amazonian Peru. Am. J. Trop. Med. Hyg..

[34] Tesh R.B. (1994). The Emerging Epidemiology of Venezuelan Hemorrhagic Fever and Oropouche Fever in Tropical South America. Ann. N. Y. Acad. Sci..

[35] Scachetti G.C., Forato J., Claro I.M., Hua X., Salgado B.B., Vieira A., Simeoni C.L., Barbosa A.R.C., Rosa I.L., de Souza G.F. (2024). Re-emergence of Oropouche Virus Between 2023 and 2024 in Brazil: An Observational Epidemiological Study. Lancet Infect. Dis..

[36] Moreira H.M., Sgorlon G., Queiroz J.A.S., Roca T.P., Ribeiro J., Teixeira K.S., Passos-Silva A.M., Araújo A., Gasparelo N.W.F., Dos Santos A.dO. (2024). Outbreak of Oropouche Virus in Frontier Regions in Western Amazon. Microbiol. Spectr..

[37] Liang G., Gao X., Gould E.A. (2015). Factors Responsible for the Emergence of Arboviruses; Strategies, Challenges and Limitations for Their Control. Emerg. Microbes Infect..

[38] Naveca F.G., Almeida T.A.P., Souza V., Nascimento V., Silva D., Nascimento F., Mejía M., Oliveira Y.S., Rocha L., Xavier N. (2024). Emergence of a Novel Reassortant Oropouche Virus Drives Persistent Human Outbreaks in the Brazilian Amazon Region From 2022 to 2024. medRxiv.

[39] de Melo Iani F.C., Pereira F.M., de Oliveira E.C., Rodrigues J.T.N.R., Machado M.H., Fonseca V., Adelino T.E.R., Guimarães N.R., Tomé L.M.R., Gómez M.K.A. (2024). Rapid Viral Expansion Beyond the Amazon Basin: Increased Epidemic Activity of Oropouche Virus Across the Americas. medRxiv.

[40] Liu B.M. (2024). Epidemiological and Clinical Overview of the 2024 Oropouche Virus Disease Outbreaks, an Emerging/Re-Emerging Neurotropic Arboviral Disease and Global Public Health Threat. J. Med. Virol..

[41] Deiana M., Malagò S., Mori A., Accordini S., Matucci A., Passarelli Mantovani R., Gianesini N., Huits R., Piubelli C., Gobbi F.G. (2024). Full Genome Characterization of the First Oropouche Virus Isolate Imported in Europe from Cuba. Viruses.

[42] Naveca F.G., Almeida T.A.P., Souza V., Nascimento V., Silva D., Nascimento F., Mejía M., Oliveira Y.S., Rocha L., Xavier N. (2024). Human Outbreaks of a Novel Reassortant Oropouche Virus in the Brazilian Amazon Region. Nat. Med..

[43] Castro M.C., Lima Neto A.S. (2024). Unprecedented Spread and Genetic Evolution of the Oropouche Virus. Nat. Med..

[44] Borborema C.A., Pinheiro F.P., Albuquerque B.C., da Rosa A.P., da Rosa J.F., Dourado H.V. (1982). Primeiro Registro de Epidemias Causadas Pelo Vírus Oropouche no Estado do Amazonas. Rev. Inst. Med. Trop. Sao Paulo.

[45] Pan American Health Organization/World Health Organization Oropouche: Cases of Mother-to-Child Transmission Under Investigation in Brazil. 18 July 2024. https://www.paho.org/en/news/18-7-2024-oropouche-cases-mother-child-transmission-under-investigation-brazil.

[46] Pan American Health Organization/World Health Organization Epidemiological Alert: Oropouche in the Region of the Americas: Vertical Transmission Event under Investigation in Brazil. 17 July 2024. https://www.paho.org/en/documents/epidemiological-alert-oropouche-region-americas-vertical-transmission-event-under.

[47] Schwartz D.A., Avvad-Portari E., Babál P., Baldewijns M., Blomberg M., Bouachba A., Camacho J., Collardeau-Frachon S., Colson A., Dehaene I. (2022). Placental Tissue Destruction and Insufficiency From COVID-19 Causes Stillbirth and Neonatal Death from Hypoxic-Ischemic Injury. Arch. Pathol. Lab. Med..

[48] Roberts D.J., Edlow A.G., Romero R.J., Coyne C.B., Ting D.T., Hornick J.L., Zaki S.R., Das Adhikari U., Serghides L., Gaw S.L. (2021). A Standardized Definition of Placental Infection by SARS-CoV-2, a Consensus Statement from the National Institutes of Health/Eunice Kennedy Shriver National Institute of Child Health and Human Development SARS-CoV-2 Placental Infection Workshop. Am. J. Obstet. Gynecol..

[49] Fitzgerald B., O’Donoghue K., McEntagart N., Gillan J.E., Kelehan P., O’Leary J., Downey P., Dean J., De Gascun C.F., Bermingham J. (2022). Fetal Deaths in Ireland Due to SARS-CoV-2 Placentitis Caused by SARS-CoV-2 Alpha. Arch. Pathol. Lab. Med..

[50] Schwartz D.A. (2017). Viral Infection, Proliferation, and Hyperplasia of Hofbauer Cells and Absence of Inflammation Characterize the Placental Pathology of Fetuses with Congenital Zika Virus Infection. Arch. Gynecol. Obstet..

[51] Schwartz D.A., Mbala-Kingebeni P., Patterson K., Huggins J.W., Pittman P.R. (2023). Congenital Mpox Syndrome (Clade I) in Stillborn Fetus After Placental Infection and Intrauterine Transmission, Democratic Republic of the Congo, 2008. Emerg. Infect. Dis..

[52] Pittman P.R., Martin J.W., Kingebeni P.M., Tamfum J.M., Mwema G., Wan Q., Ewala P., Alonga J., Bilulu G., Reynolds M.G. (2023). Clinical Characterization and Placental Pathology of Mpox Infection in Hospitalized Patients in the Democratic Republic of the Congo. PLoS Negl. Trop. Dis..

[53] Mbala P.K., Huggins J.W., Riu-Rovira T., Ahuka S.M., Mulembakani P., Rimoin A.W., Martin J.W., Muyembe J.T. (2017). Maternal and Fetal Outcomes Among Pregnant Women with Human Monkeypox Infection in the Democratic Republic of Congo. J. Infect. Dis..

[54] Schnirring L. Brazilian Scientists Probe Link Between Oropouche Virus and Poor Fetal Outcomes. CIDRAP, 18 July 2024. https://www.cidrap.umn.edu/oropouche-virus/brazilian-scientists-probe-link-between-oropouche-virus-and-poor-fetal-outcomes.

[55] Garcia Filho C., Lima Neto A.S., Maia A.M.P.C., da Silva L.O.R., Cavalcante R.D.C., Monteiro H.D.S., Marques K.C.A., Oliveira R.S., Gadelha S.A.C., Nunes de Melo D. (2024). A Case of Vertical Transmission of Oropouche Virus in Brazil. N. Engl. J. Med..

[56] Garcia Filho C., Lima Neto A.S., Maia A.M.P.C., da Silva L.O.R., Cavalcante R.D.C., Monteiro H.D.S., Marques K.C.A., Oliveira R.S., Gadelha S.A.C., Nunes de Melo D. (2024). Vertical Transmission of Oropouche Virus in a Newly Affected Extra-Amazon Region: A Case Study of Fetal Infection and Death in Ceará, Brazil. Scielo Prepr..

[57] Dashraath P., Nielsen K., Schwartz D.A., Musso D., Baud D. (2025). Vertical transmission potential of Oropouche virus infection in human pregnancies. AJOG Glob. Rep..

[58] World Health Organization Oropouche Virus Disease—Region of the Americas. 5 December 2024. https://www.who.int/emergencies/disease-outbreak-news/item/2024-DON545.

[59] Centers for Disease Control and Prevention Interim Clinical Considerations for Pregnant People with Confirmed or Probable Oropouche Virus Disease. 20 November 2024. https://www.cdc.gov/oropouche/hcp/clinical-care/pregnancy.html.

[60] Centers for Disease Control and Prevention Updated Interim Guidance for Health Departments on Testing and Reporting for Oropouche Virus Disease. 18 December 2024. https://www.cdc.gov/oropouche/php/reporting/index.html#:~:text=Oropouche%20virus%20disease%20clinical%20diagnostic%20testing%20algorithm&text=Based%20on%20current%20knowledge%20of,or%20Probable%20Oropouche%20Virus%20Disease.

[61] American College of Obstetricians and Gynecologists Update on Oropouche Virus and Potential Effects on Pregnancy. Updated November 2024. https://www.acog.org/clinical/clinical-guidance/practice-advisory/articles/2024/08/update-on-oropouche-virus-and-potential-effects-on-pregnancy.

[62] Romero-Alvarez D., Escobar L.E., Auguste A.J., Del Valle S.Y., Manore C.A. (2023). Transmission Risk of Oropouche Fever Across the Americas. Infect. Dis. Poverty.

[63] Srivastava S., Sharma D., Kumar S., Mali S.N., Mehta R., Apostolopoulos V., Sah R., do Socorro Souza Chaves T., Luna C., Rodriguez-Morales A.J. (2024). Pregnancy Loss, Oropouche Virus and the Lessons from Pernambuco, Brazil. Infez. Med..

